# An evaluation of the effect of equine-facilitated psychotherapy on patients with substance use disorders

**DOI:** 10.1371/journal.pone.0286867

**Published:** 2023-06-28

**Authors:** Kristýna Machová, Veronika Juríčková, Anna Kasparová, Kamila Petrová, Barbora Sládková, Ivona Svobodová

**Affiliations:** 1 Department of Ethology and Companion Animal Science, Faculty of Agrobiology, Food and Natural Resources, Czech University of Life Sciences, Prague, Czech Republic; 2 Center for Advanced Studies of Brain and Consciousness, National Institute of Mental Health, Klecany, Czech Republic; 3 First Faculty of Medicine, Charles University, Prague, Czech Republic; 4 Center of Equine Assisted Therapy of Psychiatric Hospital Kosmonosy, Psychiatric Hospital Kosmonosy, Kosmonosy, Czech Republic; University of Catania Libraries and Documentation Centre: Universita degli Studi di Catania, ITALY

## Abstract

Equine Facilitated Psychiatry and Psychology (EFPP) is a supportive non-pharmacological treatment program used in the treatment of patients with substance use disorder. The aim of this study was to evaluate a possible change in patient’s health and health-related quality of life between the first and fourth session of the EFPP program using the Assessment of Quality of Life (AQoL) and the Health of the Nation Outcome Scales (HoNOS). The Human-Animal Interaction Scale (HAIS) and a 5-point Likert-type scale was used for an assessment of patient’s mood in the experimental group. The study sample included 57 patients (39 in experimental group with EFPP and 18 without EFPP program) with substance use disorders hospitalized in the psychiatric hospital. Comparing the initial and final patients scores in the experimental group, there was a significant positive shift in three of the four domains of the HoNOS scale and in seven of the eight dimensions of the AQoL scale. HAIS significantly increased (p <0.001) in time as well as patient´s mood after every session and in long time perspective. Based on these results, we can assume that the EFPP might be one of the successful programs which may improve patient´s mood and social interactions in substance use disorders.

## Background

Equine Facilitated Psychiatry and Psychology (EFPP), as a form of Animal-Assisted Therapy (AAT), engages horses in and around the natural surroundings of the stables [1] to support client’s development of positive behavioral and emotional wellness [2]. EFPP has shown efficacy for building self-esteem, attachment, and trust in both the horse and the therapist [1, 3–5]. EFPP is one of the possible supportive, non-pharmacological approaches in the treatment of substance use disorders.

Substance use disorders are serious and chronically recurrent disorders in which the affected individual has difficulty restricting drug use, has strong motivation to use the drug, continues to use it despite the negative consequences, and experiences negative emotional and physiological states when discontinuing or decreasing the intake of the drug [6]. Substance use can also lead to disruption of family and social relationships and a significant change in the individual’s morals [7].

For all treatment approaches, it is essential to confront the patient with reality and break the denial, which is reinforced by the positive effects of the addictive substance. A comprehensive approach is used in the treatment of addiction and a combination of non-pharmacotherapeutic and pharmacotherapeutic procedures [8]. In a therapeutic environment, horses are perceived as non-judgmental beings, which makes it easier for clients to connect with them and communicate. Successful communication influences the building of the client’s self-confidence. Other important effects include a sense of accomplishment from correctly performing a task and the eventual overcoming of the initial concerns associated with working with horses. During EFPP, clients mentioned similar feelings as when under the influence of drugs. It was mainly a feeling of happiness, isolation from the outside world, and forgetting about negative things and worries. These effects could be caused by concentrating hard on the present moment, on the activity that the client is performing with the horse at a given time in a given place [4]. In addition to horses, the place where EFPP takes place also affects clients. The environment of the farm, the fresh air, and the momentary departure from the ward, together with the shared group experience, are among the important factors which, combined with the EFPP itself, form a comprehensive rehabilitation approach. The therapeutic benefit as a whole is then based on a combination of all activities, impressions, and emotions that are related to horses or their environment [9, 10]. In summary, EFPP includes healthy physical exercise, mental relaxation, self-reflection, enjoyment, and learning new skills. All these factors of therapy contribute to successful treatment [11]. Moreover, motivation, willingness to cooperate, and a positive attitude are the cornerstones for the persistence and effectiveness of institutional treatment for addiction [12, 13]. The presence of horses and the stay in their environment increase the willingness of clients with addiction to remain in treatment programs and improve the motivation to participate in other treatment activities [4, 10, 14].

The aim of this study was to evaluate a possible change in patient’s health and health-related quality of life between the first and fourth session of the EFPP program using the Assessment of Quality of Life (AQoL), the Health of the Nation Outcome Scales (HoNOS), the Human-Animal Interaction Scale (HAIS) and a 5-point Likert-type scale for an assessment of patients’ mood in the experimental group. All domains of HoNOS and AQoL are expected to improve in patients of the experimental group compared to the control group. In patient-horse interaction, we expect more desirable and less undesirable behavior in both humans and horses.

## Methods

### Participants

The study sample included 57 patients with substance use disorders hospitalized in the Psychiatric hospital in Kosmonosy (PNK) (Table 1). The experimental group consisted of 39 patients who underwent the EFPP program. The control group contained 18 patients who did not undergo the EFPP. Patients were diagnosed with mental and behavioral disorders caused by the use of psychoactive substances (dependence syndrome and mental and behavioural disorders due to use of sedatives or hypnotics or due to multiple drug use and use of other psychoactive substances; ICD-10; 2016) by psychiatrists.

**Table 1:** Frequencies of ICD-10 diagnoses.

Diagnose	Patients with EFPP	Controls
Dependence syndrome	36	15
Mental and behavioural disorders due to use of sedatives or hypnotics: dependence syndrome	0	1
Mental and behavioural disorders due to multiple drug use and use of other psychoactive substances: harmful use	0	1
Mental and behavioural disorders due to multiple drug use and use of other psychoactive substances: dependence syndrome	3	1

The sample consisted of 78,95% of men and 21,05% of women aged from 25–50 years (M = 31,87; SD = 7,42). Achieved education of the patients in this study ranged from primary to tertiary. The number of days, from the beginning of hospitalization to the beginning of the EFPP program, ranged between 7–118 (M = 47.5, SD = 32.1). From the beginning of hospitalization to the end of the EFPP program, 36–150 days elapsed (M = 87, SD = 33.3).

Most respondents were hospitalized for the first time (59%). At least some previous experience with EFPP was mentioned by 21.1% of patients, 23.7% had never heard of it before. The largest percentage of patients (41%) stated that they had been in contact with a horse rarely, 17.9% of patients had never been in contact with a horse. 87.2% of patients own or have owned an animal in the past, 12.8% have never had an animal at home.

### Procedure and study design

The research took place on the premises of the Kosmonosy Psychiatric Hospital–Psychiatrická nemocnice Kosmonosy (PNK). The horse center at the PNK is a center of practical training and recommended Equine Facilitated Therapies (EFT) accredited by the Czech Equine Facilitated Therapy Association. All patients enrolled in the study were informed of the course of the study beforethe commencement of the EFPP and signed an informed consent form to participate in the study. The study was approved by the ethics committee of Psychiatric hospital Kosmonosy. Data collection took place over a period of one year, from January 2019 to March 2020, with a summer break of two months and with another month-long break during the Christmas season for the therapeutic horses to rest.

The study involved patients hospitalized with substance use disorders. Data were collected during 4 weeks in each group of EFPP. Every experimental group usually consisted of 6–10 patients. In cooperation with the psychiatrist, the HoNOS scale was filled out before the beginning of the EFPP program. Before the program started, the patients themselves also filled out the AQoL scale after being instructed on how to do so. The examiner evaluated the patient-horse interaction (HAIS) in the 1^st^ and 4^th^ weeks of EFPP.

At the beginning of the EFPP, patients were asked for their demographic data. They then filled out general information about the length of their hospitalization, the number of times they had been hospitalized before, and the diagnoses they were given. They also stated whether they already had any experience with EFPP, if they ever had general experience working with horses or equestrianism, and if they had an animal at home or have had one in the past. Both the patients and the therapist evaluated patient´s mood before and after EFPP every week. After the program ended the HoNOS and AQoL scales were filled out again, using the same procedure as at the beginning of the study.

In the control group, the procedure was the same as in the experimental group except for measuring mood and filling out the HAIS scale.

### Methods and measures

#### EFPP program

The EFPP program took place once a week in seven-week shifts. Data in this study were collected throughout the year 2019. There is a monthly break between shifts in the summer months to allow horses some rest from the program and 14 days off is also included in the winter. These pauses are always included only after the end of the entire shift. The average length of one EFPP session was 120 minutes. The lessons took place under the guidance of a therapist with the assistance of two horse handlers. All three also acted as horse drivers and assisted clients in all the performed activities, which they first explained and demonstrated to them.

Activities of the individual session of EFPP were involved gradually according to the level of difficulty and the development of the therapeutic relationship with the horse. First, the patients were introduced to horses, then they gradually learned to work with the horse, first on a leash and then in freedom, and the whole cycle was completed by independent riding on a saddled horse. At the beginning of each session, a group session with the therapist took place in the interior of the stable, where patients were instructed about the course of the program and communicated their current feelings and problems, or progress in treatment. Then the therapist together with the handlers divided the activities and horses among the participants of the lesson, considering the requirements of the clients regarding the preferences of individual horses or activities. Clients worked in 2–3 member groups with one horse, with which they took turns one by one. At the end of each session, clients evaluated the content of the session, described their feelings and impressions or new experiences and skills.

The main task of the first session was to get the patients acquainted with EFPP and its course and to get acquainted with the individual horses with which patients will be working during the program. They had the opportunity to learn that each animal is an individual and that it also has its specific past, needs, pleasures, and whims. The second lesson consisted mainly of caring for the horses and leading the horses around on a leash. During this lesson, clients were able to gain confidence and acquire new skills in handling such a large animal. Participants may be provided the opportunity to adjust intention, behavior, and body language because of observing the non-verbal ‘feedback’ from the horse [15]. During the third lesson, the clients, if they agreed, had the opportunity to ride on an unsaddled horse wearing a harness with handrails. One client, under supervision, always led the horse while another client rode it. Therefore, the clients had to perceive their mutual needs and trust each other. In the fourth session, clients worked with a horse from the ground in the riding arena or the riding hall. The aim of the exercises was to control the horse without words or physical commands, only with the help of body language and gentle touches. The first exercise was the so-called quiet standing, where the client strokes the horse’s body, while both relax and connect with each other. After this interconnection, the clients tested whether the horse would follow them without commands or choose their own path. They led the horses on a sagging leash, trying to stop, start walking, and change direction without looking back at the horse or without any commands with their voice or the leash, which was supposed to remain sagging the whole time. The last exercise was to attract the horse to oneself and then drive them away from one’s personal space. The exercise was performed again without voice commands or pulling the leash. Only through body language, looks and gestures. If the client could not manage to get the horse to walk, they could use gentle touches to help. There was, of course, praise after each performed command. Clients thus had the opportunity to get to know their boundaries better, as well as the effects of indecisive or too emphatic or, on the contrary, diffident conduct. Horses are known for providing clear boundaries with approach/avoidant/assertive behavior in response to others in their environment [16]. During the fifth lesson, the clients tried to lead horses on two training leads at the same time. This way of controlling a horse resembles driving a horse harnessed in a carriage, which is controlled by means of reins. In addition to training leads, clients also had to use strong and understandable voice commands to control the horse. The patients learned to express their request effectively and adequately. During the sixth lesson, patients had the opportunity to practice riding a saddled and bridled horse and to guide the horses on their own with the help of commands. Thus, they experienced success and harmony with the animal, which voluntarily submitted to their sensitive treatment, knowing that this work was consciously voluntary by the animal. At the final lesson, clients rode saddled horses on a trip outside the psychiatric hospital. The clients were divided into pairs or groups, decided on who would ride first and mounted their horses from steps. After the first round of clients all mounted their horses, the group of horses with riders, handlers, the therapist, and the remaining clients, who were on foot, took a walk outside the hospital complex. The clients controlled the horses themselves, without the help of the handlers or the other clients. Clients who did not feel ready to ride on horseback took a little pony with them for a walk on a leash. During the trip, clients took turns both in the saddle and leading the pony in their specific group, so that everyone could get a turn.

#### Scales

*The Assessment of Quality of Life (AQoL)*. The Assessment of Quality of Life [AQoL; 17, 18] is a health-related quality of life measurement and it provides a descriptive system for a multi-attribute utility instrument, so that the scores can be used in cost-utility evaluations. The assessment tool evaluates several dimensions–Independent living, Pain, Sense (physical) and Mental Health, Happiness, Coping, Relationship, Self-worth (psycho-social). The applied version consists of 35 questions and the quality of life is demonstrated in 8 dimensions. The result is usually considered to be the so-called unweighted overall score, ranging from 0 to 100, where a higher value indicates a better result. It is a self-assessment scale, so patients are instructed on how to proceed properly before they start filling in the scale and they filled it out the same day they received it.

*Health of the Nation Outcome Scales (HoNOS)*. The Czech version [19] of the Health of the Nations Outcome Scales [HoNOS; 20] was used to evaluate the health status of working-age adult (18–65 years) patients. The instrument is constructed for an assessment of symptoms as well as social functions. It is suitable for evaluating the health status of patients/clients in the routine practice of mental health care services and monitoring the results of treatment. The assessment tool consists of 12 items with 5-point severity scales (0 = no problem 1 = minor problem requiring no action 2 = mild problems but definitely present 3 = problem of moderate severity 4 = severe to very severe problem) rated by an external evaluator, in our case a psychiatrist. Higher scores indicate more severe problems. The Czech adaptation’s reliability and validity have been proven to be satisfactory [19]. The HoNOS 12-item can be divided into four subscale scores (Behaviour, Function, Symptoms and Social) [21]. Each scale measures several problems that usually occur in clients of mental care facilities. The most severe problems that occurred within two weeks since the observation period are evaluated. The examiner was trained individually and their first few ratings on the HoNOS were checked by the trainers to ensure consistency and accuracy.

*Human-Animal Interaction scale and mood evaluation*. The Czech version of the Human-Animal Interaction Scale [HAIS; 22] was used to evaluate the extent of patient-horse interactions during EFPP. HAIS is a 24-item scale rated by an independent observer on a scale of 0–4, with 0 indicating that the item did not occur at all, and 4 indicating it occurred a great deal. HAIS consists of two subscales. First part of the scale–the Human Behavior subscale–evaluates the degree of interactions from the client towards the animal. The second part, on the other hand, evaluates the degree of interaction of the animal towards the client. These interactions "Desirable items" and "undesirable items". The total score is then the sum of points from both subscales [22, 23]. Higher total scale indicates a greater quantity of human-animal interaction, the score can range from -24 (the most negative interaction) to 76 points (the most positive interaction). In the case of this study, it was not possible to include “Animal Photography” because it is not possible to take a mobile phone or camera with you to the EFPP. The maximum score was therefore 66 points.

The observer always filled out the HAIS after the end of the first and fourth session. The observer was always the same person at all the observations. Before the beginning of the study, the observer was instructed on how to evaluate the individual items on practice observations. The administration of the project followed the instructions issued by the authors of HAIS [23].

The last monitored parameter was the mood of the patients. It was assessed before and after each EFPP session. This evaluation was performed using a Likert-type scale on a scale [24, 25] of 1–5, where 1 point = worst mood and 5 points = best mood. This assessment was performed by both the patient and the therapist who led the EFPP sessions.

#### Data analyses

Data were analyzed using statistical software IBM SPSS Statistics 25 and Jamovi 1.6.3. The analyses of HoNOS and AQoL were focused on the evaluation of between- and within-group differences. Non-parametric statistical analysis was used due to data distribution, based on the Shapiro-Wilk Test. The internal consistency of the scale was evaluated using Cronbach’s alpha coefficient.

In evaluation of HAIS and mood, the analyses were focused on the evaluation of the effect of EFPP in repeated measures. Parametric or non-parametric methods were chosen based on the Shapiro–Wilk Test results. To calculate the differences between each EFPP, and differences between the patient´s and the psychologist’s evaluation, the paired-sample T-test was used. The internal consistency of the scale was evaluated using Cronbach’s alpha coefficient.

## Results

### Honos and AQoL

#### Between-group differences

The results of the Mann-Whitney U-test didn’t show significant differences in the measured scale nor the subscales (Table 2). However, there was no reason to expect any differences between patients at the beginning of the hospitalization. After EFPP and hospitalization, patients and controls significantly differed only in the behavioral problems domain. Experimental group reached lower scores than controls did.

**Table 2:** The results of the Mann-Whitney U-test.

Scales	M (SD) before		M-W U-test	M (SD) after		M-W U-test
	Patients with EFPP	Controls	p-value	Patients with EFPP	Controls	p-value
**HoNOS total score**	11.5 (3.13)	10.9 (2.57)	0.607	8.78 (2.78)	8.80 (1.75)	0.810
Behavioral problems	4.21 (1.36)	4.50 (0.70)	0.571	3.56 (1.21)	4.28 (0.46)	**0.008**
Impairment	1.15 (1.35)	0.95 (1.11)	0.614	0.64 (1.18)	0.39 (0.99)	0.250
Symptomatic problems	2.15 (1.16)	2.28 (1.07)	0.783	1.38 (1.14)	1.50 (0.71)	0.519
Social problems	16.5 (5.40)	19.4 (0.50)	0.197	16.7 (5.77)	19.2 (0.62)	0.350
**AQoL total score**	67.3 (10.5)	70.8 (9.78)	0.235	72.6 (11.6)	75.9 (6.55)	0.394
Independent living	84.2 (11.8)	83.7 (10.8)	0.758	87.4 (12.3)	85.0 (10.89)	0.159
Senses	74.3 (12.8)	77.4 (10.7)	0.890	77.3 (12.3)	78.3 (98.71)	0.940
Pain	75.3 (18.8)	73.2 (21.2)	0.814	77.6 (20.0)	75.9 (22.25)	0.906
Mental Health	61.4 (13.6)	66.7 (11.2)	0.186	67.1 (13.7)	70.6 (14.25)	0.179
Happiness	56.4 (17.9)	60.3 (14.6)	0.297	62.5 (17.0)	64.3 (14.78)	0.586
Self-worth	60.3 (16.3)	66.2 (18.5)	0.156	68.0 (17.5)	71.1 (17.47)	0.416
Coping	59.8 (13.1)	62.7 (13.5)	0.410	69.4 (13.3)	68.6 (14.30)	1.000
Relationships	69.6 (13.7)	75.1 (11.7)	0.221	72.8 (16.1)	75.9 (10.17)	0.173

Within-group differences

The results of the Wilcoxon signed-rank test showed some significant improvements in both groups. In the group of patients, statistically significant differences after EFPP have been found in several domains of both scales, except for social problems and pain (Table 3). The Control group showed statistically significant improvement after hospitalization only in HoNOS total score, and domains of impairment, symptomatic problems, and happiness (Table 3).

**Table 3:** Wilcoxon signed-rank test results of both groups.

Scale	Patients (p-value)	Controls (p-value)
**HoNOS total score**	**< .001**	**.003**
Behavioral problems	**< .001**	.102
Impairment	**.001**	**.008**
Symptomatic problems	**.001**	**.008**
Social problems	.554	.157
**AQoL total score**	**< .001**	.059
Independent living	**.002**	.410
Senses	**.018**	.713
Pain	.274	.260
Mental Health	**.002**	.157
Happiness	**.001**	**.032**
Self-worth	**< .001**	.098
Coping	**< .001**	.096
Relationships	**.038**	.127

Reliability was calculated using Cronbach’s alpha coefficient (Table 4). Internal consistency of the HoNOS scale was poor, so the results should be cautiously. Moreover, two items were excluded from internal consistency analyses because of zero mean and variance. On the other hand, Cronbach’s alpha coefficient showed excellent internal consistency of AQoL scale.

**Table 4:** Cronbach’s alpha coefficient of HoNOS and AQol.

	Before	After
HoNOS	0.516^a^	0.443^b^
AQoL	0.914	0.928

^a^Items “Problems with living condition” and “Problems with occupation and activities” were excluded from analysis because of zero variance.

^b^Items “Problems with hallucinations” and “Problems with occupation and activities” were excluded from analysis because of zero variance.

#### Human-animal interaction scale

The results show that there was a statistically significant increase in human-animal interactions (p <0.001) in patients participating in EFPP. The average initial total score was 23.46 points, after four weeks of EFPP the average score was 41.47 points. Interactions marked as "desirable" (p = 0.002) increased significantly, while the score in the "undesirable" items decreased (p = 0.002). The overall results as well as the evaluation results of both subscales and the results of the paired-sample T-test are summarized in Table 5.

**Table 5:** HAIS scoring at first and fourth session of EFPP.

	1. Session	4. Session	T-test
	M (SD)	M (SD)	p-value
**Human behavior**	16.08 (11.06)	29.58 (7.35)	< 0.001
Desirable behavior	16.39 (10.66)	29.61 (7.29)	< 0.001
Undesirable behavior	0.32 (0.87)	0.026 (0.16)	0.054
**Animal behavior**	7.56 (4.55)	11.79 (4.04)	< 0.001
Desirable behavior	8.82 (3.62)	11.97 (4.03)	0.002
Undesirable behavior	1.27 (2.04)	0.18 (0.58)	0.002
**Total scale**	23.46 (14.62)	41.47 (9.29)	< 0.001

In a separate evaluation of the subscale of human behavior, a significant increase in the score after the fourth session was observed (p <0.001). The increase was significant in the "desirable" items (p <0.001), no statistically significant difference was observed in the decrease of the "undesirable" items (p = 0.054). The increase was particularly noticeable in items involving direct physical contact with the animal, such as "Hug the animal", "Play with animal" and "Groom the animal".

When evaluating the animal behavior subscale, a statistically significant difference was also observed, both in the overall score of animal behavior, which showed more friendly behavior of the horses (p < .001), and in its individual parts. The score in "desirable" and "undesirable" behavior of the animals also changed significantly. In this subscale, the most significant shift was in the items "Decline or avoid interaction with the human (s)" and "Make a mess or inconvenience for human (s)", i.e., in areas that are directly affected by the client’s approach or attitude.

Cronbach’s alpha coefficient showed a good internal consistency in the human behavior subscale and the total scale in the first session (Table 6). Internal consistency of the animal behavior subscale was poor, so the interpretation should be approached cautiously. Cronbach’s alpha of the total scale and both subscales of HAIS in the fourth session was acceptable. However, in both cases several items were excluded from internal consistency analyses because of zero mean and variance. Items like “Taking pictures or video” could not be evaluated, because the use of cameras is prohibited in hospitals. Moreover, the animals were not aggressive nor made any hostile sounds, so these items were always scored with zero mean.

**Table 6:** Cronbach’s alpha coefficients of HAIS.

	1.Session^a^	4. Session^b^
Human behavior	0.850	0.779
Animal behavior	0.514	0.720
Total scale	0.823	0.723

^a^ Items „Take pictures of or with animal(s) “, „Aggression toward animal “, and „ Make unfriendly sounds “were excluded from analysis because of zero variance.

^b^ Items „ Aggression toward animal “, „ Make unfriendly sounds “, and „ Behave aggressively toward you “were excluded from analysis because of zero variance.

#### Evaluation of patient mood before and after individual sessions of EFPP

When evaluating the mood of patients in the experimental group before and after each EFPP session, a statistically significant difference was observed in each of them. In addition, the gap between pre- and post-EFPP evaluations had grown over time (Table 7).

**Table 7:** Evaluation of mood by patients and examinator before and after every session of EFPP.

Session	N	Evaluated by patient					Evaluated by examinator		
		Mean Before		Mean After		P value	Mean Before	Mean After	P value
1	38	2.95	3.95		p < 0 .001		2.77	3.79	p < 0 .001
2	38	3.05	4.10		p < 0 .001		3.00	3.92	p < 0 .001
3	38	3.18	4.18		p < 0 .001		2.97	4.05	p < 0 .001
4	37	2.95	4.43		p < 0 .001		3.00	4.30	p < 0 .001

## Discussion

When comparing differences between groups in HoNOS, a significant difference was observed in favor of the experimental group in the behavioral problems’ subscale, which includes areas such as over-activity, aggressive, disruptive or agitated behavior, intentional self-harm, and alcohol or substance abuse problems. It was the improvement in the area of alcohol and drug abuse that was the aim of the EFPP program, and it seems to have been achieved in the experimental group. In a study by Pec et al. [19], it is stated that the average score of this subscale is 1.75 (SD 0.639) in healthy population. In our case, patients had a mean score of 3.56 (SD 1.21) in the experimental group and 4.28 (SD 0.46) in the control group. Thus, both groups scored higher than the healthy population, however, the score in the experimental group was lower.

Comparing the pre- and post-treatment scores in this study, there was an improvement in three of the four domains in the experimental group, whereas the control group improved in two of the four domains. The only domain that improved compared to the control group was behavioral problems. This area has been proven to be significant by both the Between-group and the Within-group differences tests. In a study by Trotter et al. [26], which focused on comparison between Equine Assisted Counseling and classroom-based counseling in areas such as externalizing, internalizing, maladaptive and adaptive behavior in students, statistically significant improvements in 17 behavior areas were observed in the group with the equine program, whereas the control group showed statistically significant improvement in 5 areas.

Possible reasons for this improvement in the area of behavioral problems may be some of the widely described effects of EFPP in terms of communication, self-confidence, satisfaction from an accomplished goal or an improvement in the quality of life [27]. In this study, similar influences of EFPP program may have affected the positive shift in over-activity, agitation, or disruptive behavior. In general, the horse is reported to be a motivational force in a treatment [3, 26].

When comparing the pre- and post-treatment scores, the experimental group showed a significant shift in area of impairment and symptomatic problems. However, the between group difference was not observed. This area includes cognitive impairment, physical illness or disability, difficulties associated with hallucinations and delusions, problems with depressed mood and other mental or behavioral disorders. The beneficial influence of EFPP on symptoms of depression was observed e.g., in MacLean’s [28] study in adults or in the study by Signal et al. [29] in children. Corring et al. [30] described a possible positive effect of EFFP on patients with schizophrenia, which is associated with delusions and hallucinations. In the case of this study, no patients with schizophrenia participated, therefore the improvement in area of symptomatic problems was in items related to depressed mood and other mental or behavioral problems. Though, in the study by Corring et al. [30] it is stated that respondents experience the feeling of “Having fun” during the program, which is recommended to take into account, because it is not always a given and may have a positive effect on various diagnoses other than schizophrenia.

One of the subscales of AQoL is the happiness subscale, which in our study showed a significant shift in both groups when evaluating the pre- and post-treatment scores. Unfortunately, for the control group, this is the only item assessed by AQoL in which a significant improvement was noted. On the other hand, in addition to the happiness subscale, a significant improvement in area of independent living, senses, mental health, self-worth, coping and relationships was observed in the experimental group. In the verbal evaluation, patients describe the EFPP program as relaxing, stating that "I have the opportunity to forget for a while and enjoy the moment", "no one judges me", "I have achieved success" or "I don’t have to analyze my situation all the time and I can relax for a while”. Very interesting was the fact that even patients who did not achieve a high HAIS score later verbally evaluated the interaction very positively, which probably stems from the more introverted tuning of the given patient. Kern-Godal, Brenna, Kogstad, et al. [4] noted that to respondents the EFPP meant “a nice break from the day” or “having an enjoyable thing to do “, they stated that “…it’s fun to ride, it is less boring than just sitting in a room”. For others, a stay in the stable was associated with a specific and positive effect „I can feel it, that it is very positive for me”. Similar opinions were expressed also in oral interviews by respondents in this study and although they were not recorded numerically, they might provide better understanding of the positive shift in the experimental group respondents.

In a study by Kern-Godal, Brenna, Arnevik and Ravndal [31] respondents reported not just feelings of happiness, but also deeper feelings and observations, such as “they don’t talk about drugs”, and other problems; “they don’t ask difficult questions” and “they treat me as who I really am rather than as a patient”. The authors pointed out participants’ self-perception of being a “person” in the stable, in contrast to being a “patient” in their usual treatment location, with participants seemingly sharing an idea of “the real me”. This may be an important factor in further treatment of substance use disorders. Participants described EFPP as a “break from usual treatment”, which is interesting, given the goal-oriented therapeutic process was still ongoing, even though the EFPP program was perceived as a “break”. Break might be viewed as novelty and this can increase attention, memory and promote behavior change. It seems though, that this form of therapy is so non-invasive that it often occurs without noticing.

This internal involuntary process is connected also to the nature of EFFP, which provides less verbal therapeutic environment (as compared to the verbal and enclosed atmosphere of the therapy room) and it is also regarded as more beneficial for some clients [32]. Several changes, feelings and realizations occur in an internal, inner sphere, where communication and a relationship between the client and the horse also take place.

The results of HAIS scores, in experimental group, which evaluate the Human- Animal Interaction showed that during the EFPP program, the degree of interaction between patients and horses increases. "Desirable" items increase over time and "undesirable" items decrease. Compared to the initial average score of the first session of EFPP, the score of the forth session almost doubled. The cause of this growth could be explained by the increased communication with horses, therapists and other participants in group therapy, the exhibited close physical contact with animals, the gradual forming of an emotional relationship with horses, the exhibited altruistic behavior, the cooperation with therapists, or an active involvement in the therapeutic program through a positive relationship with the horses involved in the program. All of these are associated with a significant improvement in mood after each session of EFPP.

The equine therapy center is freely accessible to patients from the examined ward during the entire period of their hospitalization, but the program is always attended by experimental group. Although some patients only started the EFPP program 30 days after hospitalization, the initial scores of all respondents were very similar, as was the increase in their scores over time. Thus, although patients were able to visit the horses before the program and form a bond with them, they did not seem to have done so on their own, and this positive development depends on the EFPP program–the work of the psychologist and program’s team, as well as the guided program and active and structured involvement of the client in working with the horse. The study by Templin et al. [33] states that the mere presence of cats on a ward has a positive effect on patients. Patients living in the presence of a cat were also more satisfied with their treatment outcome and recommended the clinic more. Moreover, they rated their recreational opportunities, the common rooms, and the collaboration with their primary nurse, social worker, other therapists, and psychologists significantly better, whereas there was no effect regarding the collaboration with the doctor. In a study by Beck et al. [34], they state that the mere presence of birds in therapy increases attendance and participation in sessions. However, it seems that the active involvement of the patient in the interaction with the animal within a structured, regular, and guided program can bring more significant results.

The positive shift seen in the difference in score between the first and fourth session of EFPP indicates active patient involvement and collaboration, as reported by Wesley et al. [35]. In a study where dogs were involved in the therapeutic process, respondents reported a more positive opinion of the therapeutic alliance than did individuals in the groups without the therapy dog. Clients seeking treatment for alcohol dependence reported that AAT did improve the therapeutic alliance; however, the result was not significant. Increasing the therapeutic alliance can significantly improve the probability of recovery success for clients seeking treatment for substance dependence. The quality of the alliance and not the technique or theory is the most predictive of recovery outcome success [13]. Therefore, a client receiving AAT is more likely to benefit from the treatment process, have higher retention rates, and less frequent episodes of drug and alcohol relapse [12]. A study by Kern-Godal et al. [36] shows that those who participated in the HAT program remained in treatment for a significantly longer period and were more likely to complete their program of treatment. Therefore, the degree of therapeutic alliance alongside HAIS and their correlation could be the subject of further studies. The increase in positive interaction with horses resulting from this study indicates the achievement of therapeutic goals and could therefore be related to therapeutic alliance.

In a study by Grajfoner et al. [37], they state that the presence of a handler alongside the animal appeared to have a negative, and specific, effect on participant mood, with greater positive shifts in mood being witnessed when participants interacted with the dog alone, than when interacting with both the dog and the handler. In the case of EFPP, where instructions are given at the beginning of the lesson and during the lesson communication takes place only between the horse and the client (or between two patients in case they share the horse), gives patients a greater sense of independence and confidence than if a therapist and the horse handlers were with the client at all times. In this way, they ensure a safe and correct course of action, and provide ongoing feedback or correction, but there is more space for the humans and horses themselves. This can also lead to a rapid increase in emotional and physical closeness between the patient and the horse.

The EAAT program experience, as mentioned before, can be perceived as enjoyable and fun. The study by Lemke et al. [27] studied children with physical disabilities, but their findings are consistent with the mood assessment reported by adult patients in this study, who were demonstrably in a statistically significantly better mood after each lesson. Good attunement of clients influences them and on their openness to the overall therapy and staying in treatment. Furthermore, their mood can also affect the therapists themselves and the associated effectiveness of treatment. Therapists’ affect became more positive when clients were initially positive and when clients became more positive over the session and became more negative when clients were initially negative and when clients became more negative over the session. Moreover, when therapists were initially positive in affect and when therapists became more positive over the session, clients rated the session quality to be high. Positive affect change was attributed to collaborating with the client, perceiving the client to be engaged, or being a good therapist [38]. Good client attunement can therefore also affect the course of subsequent therapies and their effectiveness.

## Limitations

A low number of participating patients is one of the limitations of this study, though this is given naturally by a practical realization of the EFPP program in a clinical setting. Unfortunately, patients with substance use disorder often leave the three-month program before completion and those patients, who had left before participating in the fourth session, where excluded from this study. The same problem also affected patients in the control group, where it was even more noticeable. A comparison of effects of EFPP programs on treatment retention appears to be a suitable topic of further study. Another possible limitation is a different number of days from the beginning of hospitalization until joining the EFPP program, which varied between patients. However, this is also due to clinical practice, as clients can be included in the program only after the previous EFPP group finishes it and only when it is indicated by a doctor. Though, both groups appeared to be comparable when reviewing the initial scores, and although respondents were free to walk around and observe the horses, the shift in their final scores seems to have been influenced only by active involvement of the horses and therapeutic work.

However, this could be also influenced by a variety of possible confounding factors that were not examined (such as, for example the “honeymoon” effect of a new program, the outdoor environment or the ambience around the stables). Of course, the EFPP program does not consist only of work with horses, but also includes other positive influences, such as the surroundings of the outdoors or of the stable. Nonetheless, it is possible that the results could differ in an unsuitable environment with the involvement of unsuitable horses, therapists, or a program composition.

## Conclusion

The main area that was improved significantly in the experimental group was the domain of Behavioral problems, which also includes difficulties with substance abuse. Comparing the pre- and post- treatment scores showed improvement in terms of HoNOS in three of the four domains and even in the overall score. In terms of the AQoL questionnaire, a significant improvement was noted in seven of the eight dimensions and in the overall score, also. As for the control group, the overall score and the two subscales of HoNOS improved, while the AQoL questionnaire showed a positive shift in only one subscale and the overall score did not improve.

The EFPP program in experimental group has led to an increase in horse-patient interactions. Compared to the initial score, the score after 4 weeks almost doubled. This is also related to a statistically significant improvement in patient mood after each EFPP session. The positive mood of patients, their motivation and willingness to cooperate could have a fundamental influence on their remaining in a long-term addiction treatment program. EFPP appears to be a possible add-on program that may affect remaining in the program and treatment success.

## Supporting information

S1 Data (XLSX)

## Abbreviations

EFPP: Equine Facilitated Psychiatry and Psychology
PNK: Psychiatric hospital in Kosmonosy
AQoL: The Assessment of Quality of Life
HoNOS: The Health of the Nation Outcome Scales
HAIS: The Human-Animal Interaction Scale
